# Extracellular vesicle therapy for obesity-induced NAFLD: a comprehensive review of current evidence

**DOI:** 10.1186/s12964-023-01292-0

**Published:** 2024-01-09

**Authors:** Jiali Zhang, Baochen Ma, Zixu Wang, Yaoxing Chen, Chengzhong Li, Yulan Dong

**Affiliations:** 1 National Key Laboratory of Veterinary Public Health and Safety, College of Veterinary Medicine, China Agricultural University, Beijing, 100193 China; 2 China Animal Husbandry Group, Beijing, 100070 China; 3 Department of Horticulture and Landscape Architecture, Jiangsu Agri-Animal Husbandry Vocational College, Taizhou, 225300 People’s Republic of China

**Keywords:** NAFLD, Exosomes, Extracellular vesicles, Nanomaterials, Therapy

## Abstract

Non-alcoholic fatty liver disease (NAFLD) as a chronic disease especially in Western countries, is still a tough question in the clinical therapy. With the rising prevalence of various chronic diseases, liver transplantation is expected to be the most common therapy after the next 10 years. However, there is still no approved drug for NAFLD, and targeted therapy for NAFLD is urgent. Exosomes as a kind of extracellular vesicle are cell-derived nanovesicles, which play an essential role in intercellular communication. Due to complex cell–cell interactions in the liver, exosomes as therapeutic drugs or drug delivery vesicles may be involved in physiological or pathological processes in NAFLD. Compared with other nanomaterials, exosomes as a cell-free therapy, are not dependent on cell number limitation, which means can be administered safely in high doses. Apart from this, exosomes with the advantages of being low-toxic, high stability, and low-immunological are chosen for targeted therapy for many diseases. In this review, firstly we introduced the extracellular vesicles, including the biogenesis, composition, isolation and characterization, and fundamental function of extracellular vesicles. And then we discussed the modification of extracellular vesicles, cargo packing, and artificial exosomes. Finally, the extracellular vesicles for the therapies of NAFLD are summarized. Moreover, we highlight therapeutic approaches using exosomes in the clinical treatment of NAFLD, which provide valuable insights into targeting NAFLD in the clinical setting.

**Graphical Abstract:**

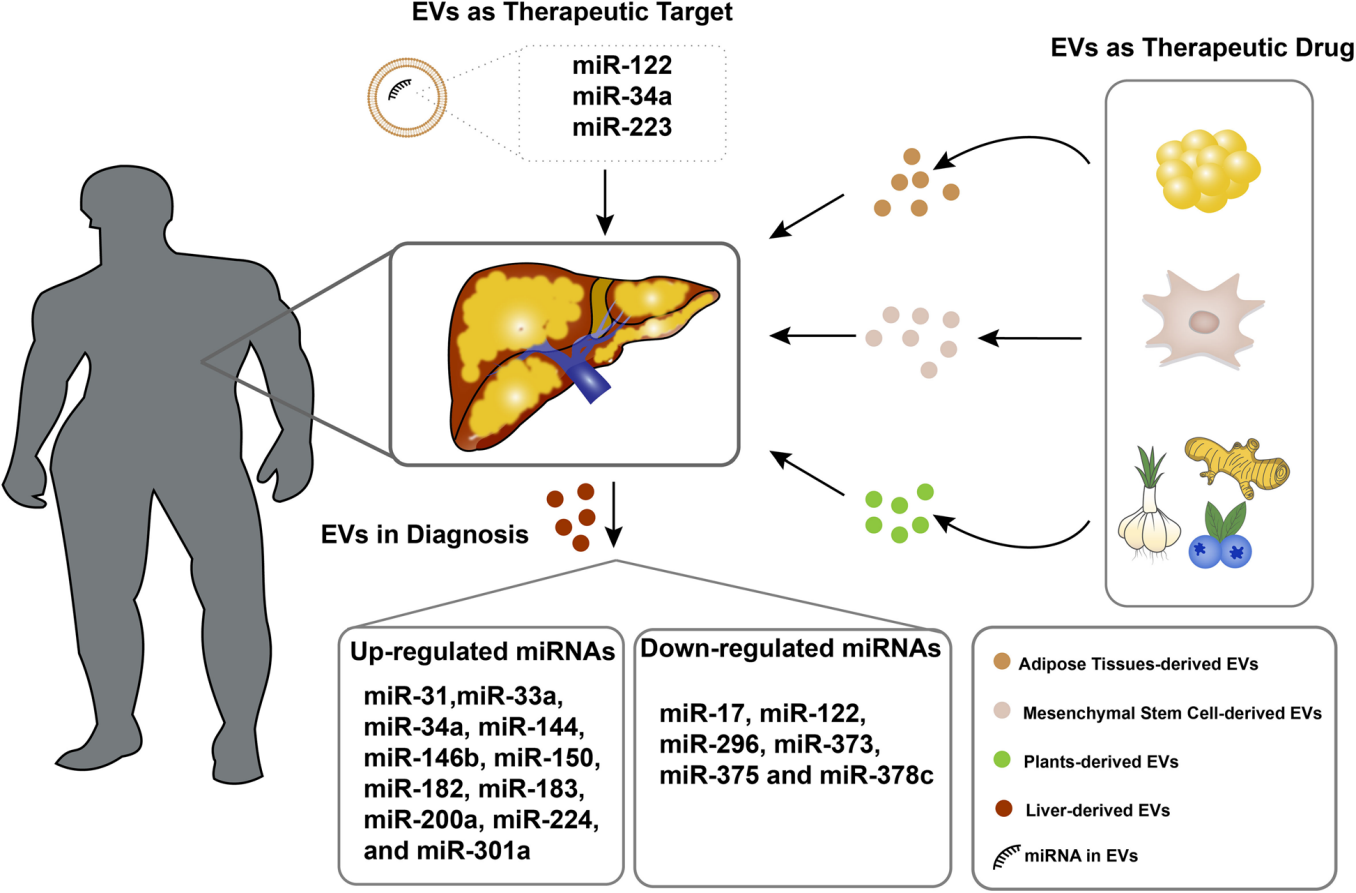

Video Abstract

**Supplementary Information:**

The online version contains supplementary material available at 10.1186/s12964-023-01292-0.

## Introduction

Obesity is a globally concerning issue with potential long-term economic burdens. Obesity can lead to a series of metabolic syndromes such as type 2 diabetes mellitus (T2DM), non-alcoholic fatty liver disease (NAFLD), dyslipidemia, inflammation, hyperglycemia, and hypertension [1]. NAFLD indicates steatosis in > 5% of hepatocytes and is strongly associated with obesity. As NAFLD develops, it can progress to Non-alcoholic steatohepatitis (NASH) and eventually to cirrhosis.

### NAFLD global prevalence statistics

Moreover, obesity prevalence varies by ethnicity, country, gender, and age, and it tends to be higher than in the general population [2]. Despite having a lower BMI, Chinese individuals face a higher risk of NAFLD due to hereditary factors. The incidence of NAFLD in China has increased to 5.2% by 2016. In addition, the incidence of NAFLD has broken out among the younger generation in China [3]. Australia has a high prevalence of overweight individuals and is known for the common occurrence of NAFLD, with a prevalence of approximately 20–30%. In contrast, NAFLD prevalence is relatively low in Africa. Besides, Hispanics have the highest prevalence of NAFLD, and African Americans have the lowest. Regarding age and gender, older individuals exhibit a higher prevalence of NAFLD and NASH, while men are more prone to NAFLD compared to women [4]. Moreover, NAFLD can progress to NASH and cirrhosis, potentially resulting in mortality or necessitating liver transplantation [5].

### The pathogenesis of NAFLD

**Fig. 1:**
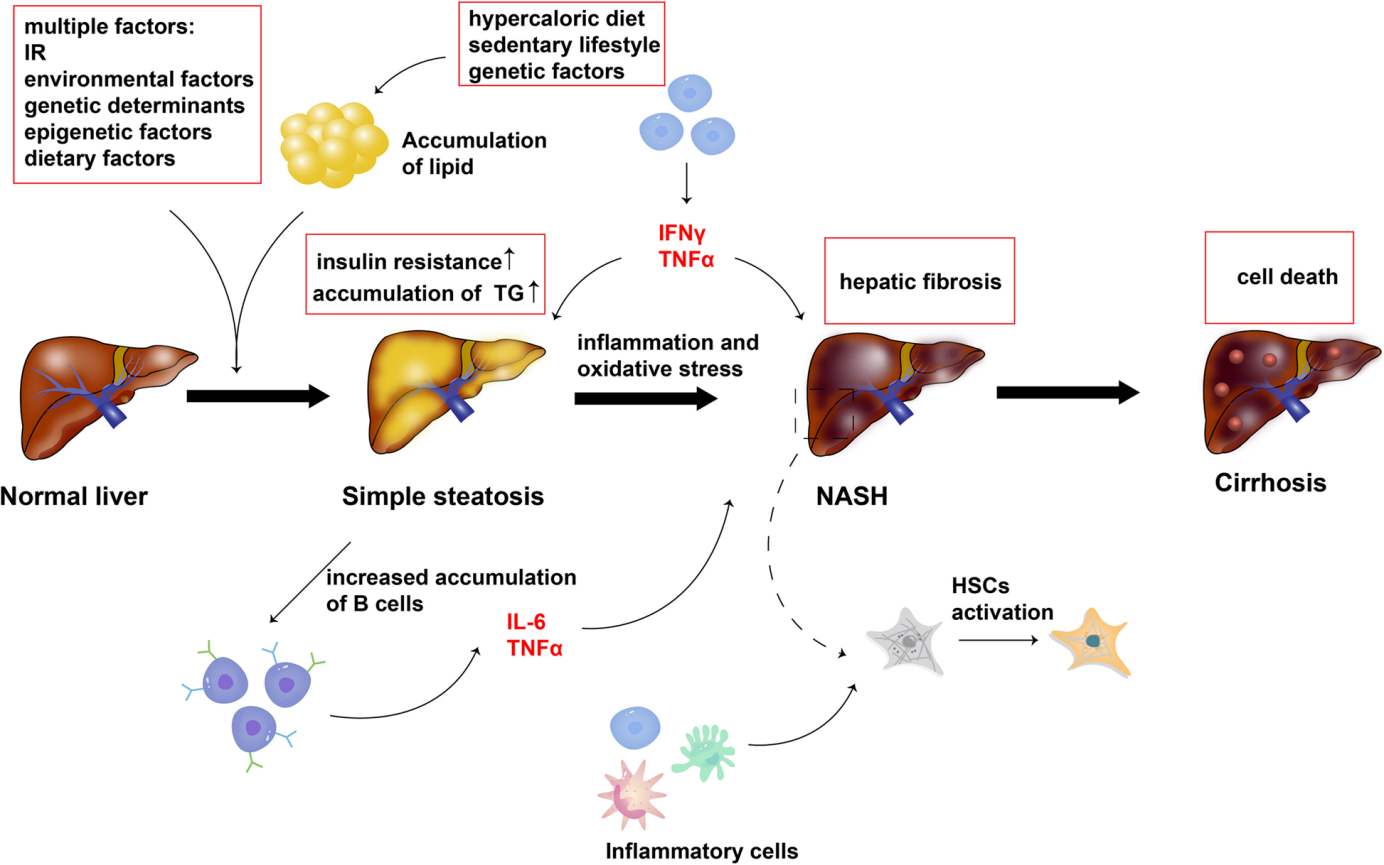
Multiple factor and the role of immune cells in the pathology of NAFLD

However, the pathogenetic mechanism of NAFLD is not clear, and the firstwidely accepted is the “two-hit” theory. Hepatic accumulation of triglycerides and insulin resistance due to hypercaloric diet and sedentary lifestyle or genetic factors lead to the “first hit”. Then comes the “second hit”, which can trigger inflammation and oxidative stress under fatty liver deposits, eventually leading to liver fibrosis and even cirrhosis [6]. However, the “two-hit” theory is outdated because it cannot explain the changes in glucose and lipid metabolism in NAFLD. Therefore, the “multiple-hit” theory has been introduced. Multiple factors act together to induce NAFLD, such as insulin resistance, environmental factors (shift work, changes in commensal microbiota), genetic determinants (PNPLA3, TM6SF2), epigenetic factors (overeating, obesity and starvation) and dietary factors etc. [7]. In addition, some immune cells also play an important role in the development of NAFLD, such as CD8^+^T cells can secrete IFNγ and TNFɑ, and B cells are likely to accumulate in the liver leading to NASH through the secretion of IL-6 and TNFɑ [8].Therefore, the development of NAFLD is not simply the accumulation of lipids in the liver, but rather the joint action of internal and external factors (Fig. 1).

Under the function of accumulation of lipid and other multiple factors, IR and TG increased, leading to simple steatosis. B cells then accumulated in the liver and secreted pro-inflammatory factors (IL-6 and TNFɑ), which could promote liver fibrosis. Inflammation and oxidative stress also promoted NASH. At the same time, T cells secreted IFNγ and TNFɑ, which played an important role in both simple steatosis and NASH. As the inflammation progressed, the inflammatory cells activated hepatic stellate cells (HSCs), which was the key to liver fibrosis. The next step was cell death leading to cirrhosis.

### Clinical approaches to NAFLD treatment

Currently, strategies like exercise and dietary interventions, such as the Mediterranean diet (MD) [9], ketogenic diet [10], are used to treat NAFLD. Exercise activates the AMPK/ULK1 pathway to enhance lipophagy, while dietary intervention inhibits the Akt/mTOR/ULK1 pathway to enhance lipophagy [11]. However, adherence to these interventions can be challenging for some individuals, highlighting the importance of drug intervention. Currently, there are no approved drugs specifically for NAFLD treatment. Although several anti-obesity and anti-diabetic drugs are used in clinical practice, their misuse can lead to acute liver injury and severe chronic liver disease [12]. Moreover, certain drugs like pioglitazone, an anti-diabetic medication, can induce various side effects, including weight gain, peripheral edema, congestive heart failure, bone fracture, macular edema, and anemia [13]. Targeted therapy for the liver is therefore particularly important.

### EV and NAFLD

Extracellular vesicles (EVs) are a kind of nanosized vesicles secreted by various cells. As a communication tool, they carry transmembrane proteins, which is a good material for intercellular communication. The biogenesis of EVs plays a crucial role in the treatment and diagnosis of diseases. Assessing the secretory capacity of EVs can help uncover the interrelationship between autophagy and paracrine secretion in CD146 + cells, offering new insights for cardiac regenerative therapy in myocardial infarction [14]. In addition, EVs biogenesis may be associated with inflammation. It has been shown that croup promotes exosome biogenesis [15]. Conversely, it has been shown that water-soluble multifunctional polyhydroxylated-polyhedral oligomeric silsesquioxane (POH-POSS) induces angiogenesis while promoting exosome secretion [16]. As EVs in the liver, they can be used as a communication tool for the pathology of NAFLD. In the liver, exosomes transport different miRNAs that may be used as biomarkers or therapeutic agents in NAFLD [17]. Other Research shows that those with NAFLD had higher levels of exosomes and even liver-derived exosomes in plasma than healthy controls. DNA damage-regulated autophagy modulator (DRAM) is a lysosomal protein and is regulated by TP53. As one of the genes associated with exosome secretion in patients with NAFLD, the level of DRAM was higher in liver tissue from patients with NAFL than from healthy controls. Lipid-induced DRAM can recruit stomatin (STOM) to lysosomes, leading to lysosomal membrane permeabilisation (LMP) and further promoting exosome release from hepatocytes. While silencing or down-regulating DRAM, stomatin (STOM) could not be recruited to lysosomes and the formation of multivesicular bodies (MVBs) would be degraded, inhibiting the release of EVs [18]. In the therapeutic field, modified MSC-derived exosomes have been shown to be effective in the treatment of cancer, central nervous system disorders, age-related diseases, and metabolic diseases such as diabetes [19–21]. In addition, EVs have many advantages, such as low immunogenicity, low toxic, and both synthetic nanocarriers and cell-mediated drug delivery [22], so they can be applied for targeted therapy. Therefore, the research for cell-free therapy is an urgent and promising therapy for NAFLD.

This review focuses on EVs therapies for NAFLD in terms of both EVs as targets and therapeutic agents, which provides new ideas and insights for treating NAFLD in the clinical setting.

## The biogenesis of EV

**Fig. 2:**
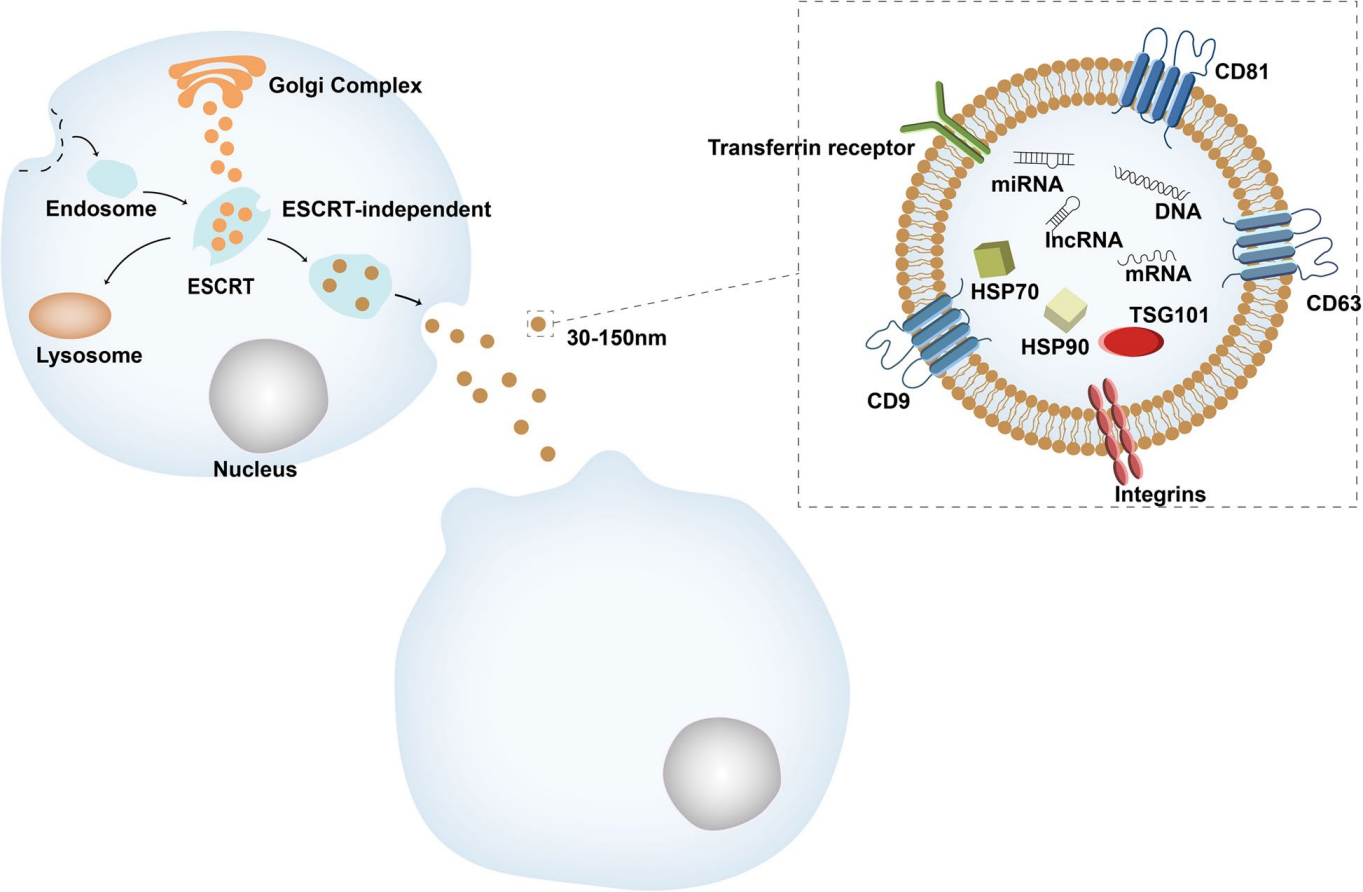
Exosome biogenesis and the structure of exosome composition

EVs are a kind of nanovesicles with a lipid bilayer. Initially considered cellular waste, extracellular vesicles (EVs) gained prominence when Neville Crawford discovered that they contain lipids and proteins [23] and can be released by almost any cell type. These vesicles are now considered to be a communication tool between cells that play an important role in the process of physiology and pathology, including immune modulation and cancer metastasis [24]. The exosomal pathway involves several steps: the formation of endosomes, MVBs also called multivesicular endosomes (MVEs), and the release of EVs [25]. The endosomal system is the starting point for exosomes. There are two pathways involved in their biogenesis: Endosomal Sorting Complex Required for Transport (ESCRT) dependent and ESCRT independent [26]. ESCRT dependent can mediate inward budding of MVEs to form intraluminal vesicles (ILVs), which are then secreted by fusion of MVEs and the cell surface [27]. And the formation of ILVs may also be independent of ESCRT, Wei et al. found that activation of RAB31 could recruit the GTPase-activating protein TBC1D2B, which could prevent lysosomes from fusing with MVEs to launch ILVs that secrete EVs [28]. Besides, there is a similar pathway to the exosome pathway called the lysosomal pathway, which involves plasma membrane invagination to form lysosomal membranes and the fusion of some ubiquitinated products to be degraded by lysosomes. However, the main difference between the exosomal and the lysosomal pathways is that the vesicles formed by plasma membrane invagination in the exosomal pathway contain more cholesterol [29] (Fig. 2).

EVs secreted by cells can be classified into three types: exosomes, microvesicles and apoptotic bodies. The diameter of exosomes is about 30–150 nm, so they can be confused with viruses (20–300 nm), microvesicles (200–1000 nm), and apoptotic bodies (800–5000 nm) [26]. There are three types of EV uptake: the first is membrane fusion, due to the lipid bilayers, exosomes can fuse directly with the recipient cell. The second is receptor (direct) interaction, because of the transmembrane proteins in exosomes, exosomes can bind to the recipient cell through the proteasomes. The last is internalization, which involves the use of common endocytic pathways [30].

## The composition of EVs

In terms of protein composition, EVs contain not only intracellular proteins but also extracellular proteins. Protein cargo selection is a complex process involving various components of the endosomal pathway and the plasma membrane, but the process by which certain proteins are sorted more efficiently than others is unclear in EVs. Identification of protein markers is one of the best way to characterise EVs. Each EV contains unique proteins, including the classical EV markers such as CD9, CD81, CD63, which show different levels in different EVs. However, Ruben et al. found that there were 6 types of EVs markers (ENO1, GPI, HSPA5, YWHAB, CSF1R, and CNTN1) and they had the same levels in EVs of almost all cells [31]. However, Annexin A1 and the glycolytic enzyme GAPDH were absent in classical EVs but present in both small and large EVs [32]. As plant-derived EVs, they usually have a low protein content. For instance, ginger-derived nanoparticles (GDNPs) contain some cytosolic proteins (e.g. actin and proteolytic enzymes) and some membrane proteins (e.g. membrane channels or transporters) [33]. Besides, exosome-like nanoparticles derived from grapes were found to contain aquaporin, heat shock cognate protein and some enzymes [34].

EVs have lipid bilayer structures, lipid profile analysis shows that PELNVs contain different lipids, and the most is phosphatidic acid (PA), and then monogalactosyldiacylglycerol (MGDG), phosphatidylcholine (PC), phosphatidylinositol (PI) etc. [35]. However, animals-derived EVs are different from PELNVs, they contain cholesterol (CHOL), phosphatidylserine (PS), phosphatidylinositol (PI), which depend on different cell types [36].

As for RNA, EVs generally carry small non-coding RNAs (snRNAs), mRNA, microRNAs (miRNAs), circular RNAs (circRNAs) and some fragmented RNA instead of a large number of RNA molecules [37]. CircRNAs play an important role in cancer proliferation and metastasis, and they are resistant to many advanced drugs, which have a promising outlook in clinical cancer therapy [38].

In addition, EVs contain DNA such as single-stranded DNA, double-stranded DNA, genomic DNA and mitochondrial DNA. However, there is currently less research in this area, so the physiological role of DNA in EVs is not well understood [37] (Fig. 2).

Invagination of the cytosol to form endosomes, and Golgi complex bud loaded vesicles and they enter endosomes leading to the formation of multivesicular bodies (MVBs). There were two pathways that could be chosen: ESCRT dependent and ESCRT independent. And MVBs can be degraded by lysosomes or fuse with the plasma membrane. The most common membrane marker proteins of EVs are CD9, CD63, CD81. A number of transferrin receptors and integrins are also found on the membrane.

## Isolation and characterization of EVs

### Methods of EVs isolation

**Fig. 3:**
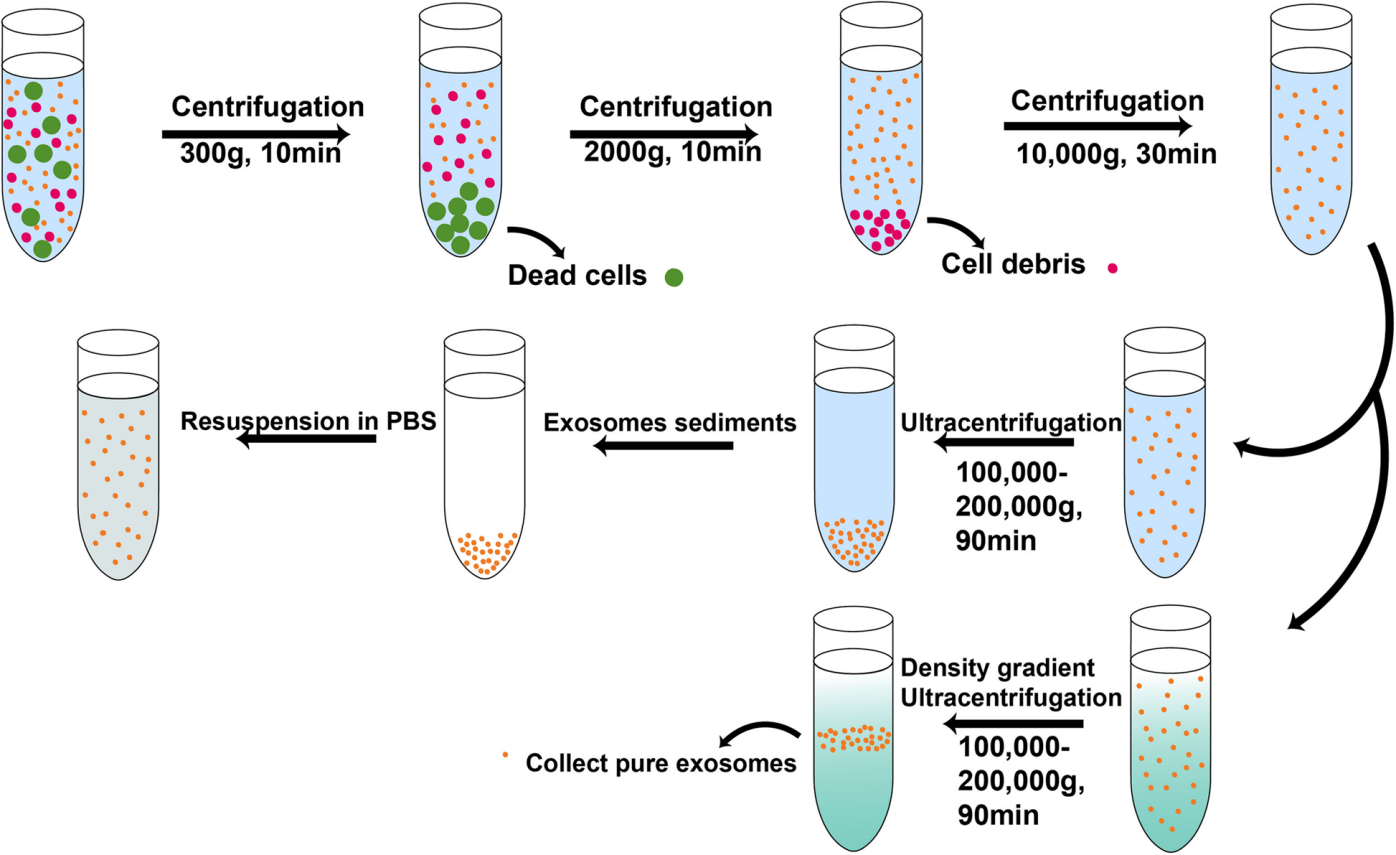
Schematic representation of isolating EVs by centrifugation, ultracentrifugation and density gradient ultracentrifugation

EVs isolation includes ultracentrifugation, filtration, chromatography, immunoaffinity isolation, membrane affinity, phosphatidylserine affinity, microfluidic techniques, polymer precipitation [39, 40]. The “gold standard” for EV isolation is ultracentrifugation. First, a low-medium speed series is used to remove dead cells and debris and even some large-sized EVs, then a higher speed with a centrifugal force of more than 100,000 × g is used to separate EVs [41]. EVs can be collected from various samples (e.g., blood, saliva, urine, nasal secretion, breast milk, cerebral spinal fluid (CSF), cultured cell-conditioned medium), and sometimes biological fluids can be diluted by PBS with an equal volume based on its viscosity, which can improve the recovery of EVs [42]. According to their buoyant density, the most commonly used method is sucrose density-gradient centrifugation (DGC), which can be used to purify exosome-like vesicles (ELVs). There are two methods: continuous and discontinuous (stepwise) gradient centrifugation. Research has shown that grapefruit-derived ELVs can be purified using sucrose gradients (8%, 30%, 45%, 60%) [43, 44] (Fig. 3). However, this method has many drawbacks, such as being very complicated, time consuming and low yield. Hence, one protocol applied a 30% sucrose double-cushion centrifugation to improve particle isolation [45]. In the field of virus research, viruses share many of the characteristics of EV such as size, structure and biogenesis. The high osmotic pressure of sucrose can damage the sample, but metrizamide, iohexol and iodixanol are very good at precipitating viruses. Research shows that iodixanol has been used to successfully separate exosomes from HIV, while sucrose was unable to separate them. Besides, iodixanol can also be used to isolate Evs that based on density, such as viscous saliva [46]. Recently, EVs have been isolated from HEK-293 [47], uterine aspirates [48], semen [49], breast milk [50], saliva [51], amniotic fluid [52], human and murine lymphoid tissues [53], bronchial fluid [54], CSF [55], tears [56], human serum [57] and human urinary [58], skin [59], which can be applied for various cancer detection. Besides, in both normal and pathological states, various cells can also secrete EVs such as endothelial cells [60], tumor cells and immune cells [61], epithelial cells [62], astrocytes [63] etc. Some food and plants also can secrete extracellular vesicles, which are defined as exosome-like vesicles. For instance, grapefruit-derived extracellular vesicles can promote wound healing [64], ginger-derived nanoparticles can protect alcohol-induced fatty liver [65], lemon-derived extracellular vesicles can induce apoptosis of gastric cancer cells [66], honey-derived EVs have antibacterial effect [67], celery-derived EVs can suppress immune cells for excessive immune response [68] etc.

First, dead cells and cell debris were removed by centrifugation. Then, the EVs sediments were resuspended in PBS by ultracentrifugation. In addition, EVs could be purified by density gradient ultracentrifugation. All the above operations were performed at 4℃.

### Methods of EVs characterization

The characterization of EVs requires the use of both physical and chemical methods. According to different sizes, they can be clearly differentiated by some physical protocols such as flow cytometry, dynamic light scattering (DLS), nanoparticle tracking analysis (NTA), transmission electron microscopy (TEM), resistive pulse sensing (RPS), atomic force microscopy (AFM) [69, 70], enzyme-linked immunosorbent assay (ELISA) [40, 71]. These protocols can be used according to the different sizes of EVs (Table 1).

**Table 1:** Techniques used to characterize microvesicles

Techniques	Diameters (nm)	Pros	Cons	Ref.
Flow cytometry	270–600	Relative size, particle granulation and high throughput	Size cannot be measured, high concentrations are not allowed, only detect biomarkers of exosomes	[69, 71–73]
DLS	Minimum:10	Diameter range, particle distribution	One sample type, not biochemical or cellular originated	[41]
NTA	Minimum: < 30	Average size, mode value and size distribution, small particles can be detected	Particle concentration required, limiting detection of fluorescent signal	[69]
TEM	Can detect the smallest vesicles	Morphology, diameter	Cumbersome manipulation, cause changes in the morphology of the EVs	[74]
RPS	Minimum: 70–100	Diameter, accuracy	Pore clogging and pore stability, time-consuming	[70]
AFM	Sub-nm resolution	Measure samples in their native conditions, the 3-D image of surface topography, diameter	Expensive samples have substrate to attach on the substrate stably	[75]
ELISA	NA	Can be quantified based on multiple extracellular vesicle surface marker proteins	Size distribution of nanovesicles could not be obtained	[71]

*Pros*: advantages; *Cons*: disadvantages; *NA*: not applicable

## The function of EVs in NAFLD

EVs can play an essential role in regulating intercellular communication because of a functional, targeted, mechanism-driven accumulation of specific cellular components in EVs. Therefore, EVs can effectively promote and restrain diseases by transporting protein, metabolites, and nucleic acids, which have an effect on immune responses, viral pathogenicity, pregnancy, some diseases, and cancer progress [76]. Once released extracellularly, exosomes can transfer vesicle contents to target cells through activation of surface receptors, vesicle internalization (phagocytosis, endocytosis), or membrane fusion [77]. The potential functions of EVs in NAFLD include two main aspects: diagnosis and as a therapeutic medicine. Here, we describe examples of the function of EVs in NAFLD.

### The function of diagnosis

In NAFLD, non-invasive tools do not fully reflect the histologic changes in the patient's liver, but those damaged EVs are better able to translate disease progression. Higher concentrations of EVs secreted by T cells and monocytes in patients with NAFLD [78]. In addition, miRNA in EVs can also be an indicator of NAFLD diagnosis. miRNAs as a small and non-coding RNA, which play an important role in the development of liver disease. Different miRNAs show different trends of change, some miRNAs express an upregulation in livers (e.g., miR-31, miR-33a, miR-34a, miR-144, miR-146b, miR-150, miR-182, miR-183, miR-200a, miR-224, and miR-301a), and others express a downregulation in livers (miR-17, miR-122, miR-296, miR-373, miR-375 and miR-378c) [79]. The expression of some miRNAs are controlled by liver- enriched transcription factors such as miR-122 and miR-194/192, and some are controlled by inflammation such as miR-223, miR-21 and miR-155, which can be promising targets for NAFLD treatment [80].

### The function of therapy

There are two main strategies for treating NAFLD: EVs as a therapeutic target and EVs as therapeutic drug.

#### EVs as a therapeutic target

miR-122 is a liver-specific miRNA and it is closely related to liver disease [81]. Research has demonstrated that knocking down miR-122 by the transfection of miR-122 inhibitor can re-activate the LKB1/AMPK axis to inhibit lipid overproduction, thereby alleviating NAFLD [82]. In addition to this, other miRNAs are also strongly associated with NAFLD progression. miR-34a is also regarded as a miRNA that associate with NAFLD. Research has shown that silencing miR-34a can directly target *PPARα* to increase the expression of *PPARα*, which induced transcriptions of fatty acid beta-oxidation factor. Meanwhile, silencing miR-34a can also decrease TG to inhibit the lipid accumulation [83]. In addition, miR-223 can also target inflammatory and oncogenic genes in the livers. Research has shown that overexpression of miR-223 can inhibit two downstream targets, Cxcl10 and Taz, in the liver, which can be thought to be a therapy for NASH [84].

#### EVs as therapeutic drug

##### Adipose tissues-derived EVs

Mammals have two types of adipose tissue: white adipose tissue (WAT) and brown adipose tissue (BAT). BAT can secrete EVs to communicate with other organs. A research showed that BAT-derived exosomes (BAT-Exos) visibly alleviated metabolic syndrome in HFD mice, and BAT-Exos targeted liver after intravenous injection which reduced two inflammatory genes (TNFα and IL1β), the level of ALT and lipid deposition in the liver. BAT-Exos can transfer the functional protein to the liver, which may play an important role in NAFLD and be treated as a promising treatment for NAFLD [85]. FGF21 (fibroblast growth factor-21) is produced in the liver and released into the circulatory system to regulate metabolism. BAT-Exos can transporter circulating miRNAs, which may act as a novel adipokine to remotely regulate FGF21 in the liver [86]. While eWAT-derived exosomes (ATEx) under ER stress activated cAMP signaling to elevate Aldo–keto-reductase1b7 (AKr1b7) protein level in hepatocytes, which enhanced hepatic lipid synthesis and eventually induced NASH [87]. In addition, BAT-Exos can cause WAT browning, which provides new therapeutic strategies for obesity-related metabolic diseases [88]. This method can overcome the difficulty of BAT application and the problem of immune intolerance. Regarding the isolation and extraction of adipocyte extracellular vesicles, in short, the adipose tissue was abbreviated to small pieces and then they were cultured in serum-free DMEM. Afterwards, the culture supernatant was taken and centrifuged in density gradient to obtain EVs [89]. However, the drawback is that it is difficult to isolate specific exosomes from mature adipocytes, so it is challenging for clinical application.

The function of WAT is storing energy, and that of BAT is dissipating energy mediated by mitochondrial uncoupling protein 1 (UCP1). In addition, some brown adipose tissues differentiated with the classical showed in WAT, which were termed “browning” or “beiging”, and the function of beige is as the same as BAT [90]. Recent studies have indicated that BAT can be activated through various means such as cold-induced thermogenesis (CIT), which triggers sympathetic neurons to release norepinephrine via activation of ADRB3. Additionally, diet-induced thermogenesis (DIT) can also activate ADRB3 and AMPK/PGC-1α signaling through the consumption of capsaicin, resveratrol, curcumin, and similar substances. Furthermore, WAT browning can be induced through exercise and the presence of certain intestinal microorganisms such as *Adlercreutzia, Mogibacteriaceae, and Ruminococcaceae* [91, 92]. When excessive caloric intake occurred, WAT would expand to accommodate more triglycerides and combat the ectopic lipid deposition that induced obesity. While obesity induced inflammation in WAT, BAT had less inflammation than WAT [93].

##### Mesenchymal stem cell-derived EVs

Stem cell-based therapy now considered a significant treatment for metabolic diseases such as type 1 diabetes, and stem cells could be induced into specific cells to repair tissues, e.g., induced pluripotent stem cells (iPSCs) or embryonic stem cells (ESCs) could directly differentiate into hepatocytes [94]. Stem cells could continuously secrete trophic factors as mediators, and stem cell-EVs as a communication tool to regulate cell fate. So far, the only stem cells capable of producing exosomes in large quantities are mesenchymal stem cells (MSCs) [95]. Mesenchymal cells are multipotent adult stem cells, also known as multipotent mesenchymal stromal cells, which can be isolated from adipose tissue, bone marrow, placenta, umbilical cord and dermis. As a regeneration medicine, mesenchymal stem cells (MSCs), a nonhematopoietic, heterogeneous population of plastic-adherent cells were widely used [96]. Since MSCs regulate tumor cells in a paracrine manner rather than a cellular manner, and MSC-derived EVs happen to operate in a paracrine manner, which makes MSC-EVs a treatment for disease [97]. It has been shown that the mitigating effect of MSC on liver disease is mainly through the paracrine pathway of MSC. In addition, research has demonstrated that MSC-derived exosomes (MSC-Exos) could be used as a cell-free therapy for hepatic regeneration in drug-induced liver injury [98]. Therefore, MSC-EVs could be a potential treatment for NAFLD.

Exosomes derived from human umbilical cord mesenchymal stem cells (Huc-MSC) have been used to treat animal models with liver disease. HSTP1-modified Huc-MSC exosomes (HSTP1-Exos) can specifically target the hepatic stellate cell (aHSC) region by fusion with enriched membrane protein (Lamp2b), which provides a new strategy for clinical liver fibrosis treatment [99]. In addition, human umbilical cord blood mesenchymal stem cell (hUCB-MSC) derived exosomal miR-124 promotes rat hepatocyte proliferation by inhibiting Foxg1 expression [100].

Exosomes secreted by adipose stem cells also exert an inhibitory effect on lipid degeneration. Human adipose-derived stem cells (hASC)-derived extracellular vesicles activate the HGF/c-Met pathway by releasing lncRNA H19, which promotes liver regeneration and reduces cell necrosis and inflammation [101]. In addition, treatment with adipose-derived stem cells (ADSCs) from eWAT secreted exosomes by intraperitoneal administration could induce M2 macrophage polarization by transporting STAT3 to express high levels of Arg-1 and IL-10, which could improve WAT inflammation and promote WAT beiging. Overall, this has been shown to be effective in improving fat production from de novo in NAFLD [102].

In acute liver injury (ALI) induced by CCl_4,_ MSC-Exos from mouse compact bones causes system XC^−^ activation to block CCl 4-induced iron death in hepatocytes by down-regulating mRNA levels of prostaglandin endoperoxide synthase 2 (Ptgs2) and lipoxygenases (LOXs) and by concomitant restoration of protein levels of SLC7A11 in primary hepatocytes and mouse liver [103]. These therapeutic approaches can be a target for the treatment of NAFLD. It can be seen that MSC-Exos play an important role in the treatment and and prevention of NAFLD, and could also alleviate the development of NAFLD.

Overall, mesenchymal stromal cell-derived exosomes have a therapeutic effect in the stages of NAFLD pathogenesis such as adipose de novo genesis, inflammatory factor infiltration, and liver fibrosis. Compared to MSC-Exos, although MSCs have differentiation, they also have low survival in damaged tissue areas. However, MSC-Exos have lots of advantages such as smaller size, lower complexity, easier to produce and longer preservation. More importantly, they can carry drugs and can be modified to be applied for targeted therapies. But there are still some challenges. For clinical use, it is necessary to continuously derive new batches of MSC from hESC to obtain a source of cells for EVs, followed by a series of operations such as isolation and purification, which are costly and will make it difficult to commercialize the production of MSC exosomes [95, 104].

##### Plants-derived EVs

Plant-derived exosomes can be described as plant exosome-like nanovesicles (PELNVs). This nomenclature has become less strict in recent years [105]. Compared to mammalian cell-derived exosomes (MDEs), PELNVs do not contain zoonotic and human pathogens, and have non-immunogenic and innocuous properties that can be used as a method of targeted drug delivery [106]. Besides, not much is known about PELNVs. Despite the limited number of articles on PELNVs for NAFLD, research in this area offers new strategies for the future treatment of NAFLD.

Research has shown that blueberry-derived exosomes-like nanoparticles (BELNs) promote translocation of nuclear factor erythroid 2-related factor 2(Nrf2), an important transcription factor for antioxidant proteins, from the cytoplasm to the nucleus, induces the expression of Bcl-2 and heme oxygenase-1 (HO-1) and reduces Bax to prevent HepG2 apoptosis. In addition, BELNs supplementation could attenuate mitochondrial oxidative stress in hepatocytes to improve liver dysfunction through increasing the activities of two important antioxidative enzymes (SOD and GSH) and inhibiting transcription factors for de novo lipogenesis (FAS and ACC1) in HFD-fed mice [107].

Ginger-derived nanoparticles (GDNP) could target liver in mice and activated Nrf2 to enhance expression of a group of liver detoxifying/antioxidant genes. This is mainly due to the fact that gingerenol in GDNP acts in a TLR4/TRIF-dependent manner in Nrf2 induction [65]. Besides, GDNP prevents insulin resistance by blocking Akt-1-mediated Foxa2 phosphorylation and restoring homeostasis of intestinal epithelial and hepatic Foxa2 signaling in HFD-fed mice. In addition, the researchers found that oral administration of GDPV prolonged the life span of mice and prevented the development of skin diseases [108].

GDV from garlic reduces inflammation (such as TNF-α, IFN-γ, and IL-1β) in the liver cells caused by LPS. This is due to the CD98 glycoprotein, which has a lot of mannose motifs and is found in HepG2 cells. CD98 helps to take in GDV by binding with lectin-type proteins. GDV has the potential to be a better treatment option for NAFLD due to the high expression of CD98 [85].

Studies have found plant miR159a and miR156c in exosome-like nut nanovesicles (NVs) that match with mammalian TNF receptor superfamily member 1a (*Tnfrsf1a*) transcripts. In addition, they have demonstrated NVs can target *Tnfrsf1a* gene to inhibit tumor necrosis factor-alpha (TNF-α) signaling pathway in adipose tissue of HFD-treated mice [87]. TNF-a increases FFA production in adipose tissue and liver and many pathological conditions are characterized by *Tnfrsf1a* dysregulation, leading to acute and chronic inflammatory processes [109]. Hence, NVs may be a promising therapeutic strategy for the treatment of nonalcoholic steatohepatitis.

Component vesicle-like nanoparticles in honey (H-VLNs) inhibit the formation and activation of inflammatory vesicles, including nucleotide binding domain and leucine-rich repeat related (NLR)-family, pyrin domain containing 3 (NLRP3), with miR-4057 in H-VLN specifically inhibiting NLRP3 activation to provide liver injury therapy. The extraction of H-VLNs removes honey's sugar content, making it partially therapeutic for diabetics [110] (Table 2).

**Table 2:** Characterization, Potential Function of Plants/food exosome-like nanovesicles related to fatty liver

Plants/Foods	Size (nm)	Targeted cell	Function	Ref.
Blueberry	150–200	HepG2 cell	-improving the distribution of Nrf2 in cells-inhibiting the expression of fatty acid synthase (FAS) and acetyl-CoA carboxylase 1 (ACC1)	[107]
Ginger	GDN: average 386.6 GDEN2: average 294.1 GDNP: 250 ± 72	Murine colon (MC-38), human colon (Caco-2) cell	-activating Nrf2 and inhibiting the production of ROS. -increasing the expression of Foxa2 to alter the composition of IEC exosomes, eventually prevents IEC exosome mediated insulin resistance	[65, 108]
Garlic	70–200	HepG2 cell	- be combined with HepG2 via CD8 and II lectin to provide an anti-inflammatory effect-	[85]
Nut	200 nm	3T3-L1, RAW 264.7 cells, peripheral blood mononuclear cells, human HEK293	-plant miR mimics in nut-derived exosomes can target Tnfrsf1a gene to reduce the expression of TNF-α, which have the effect on treating inflammatory-associated metabolic diseases	[87]
Honey	120–180	primary macrophage BMDMs	-miR-4057 in honey-derived exosomes suppress the NLRP3 inflammasome, which reduce inflammation in liver	[110]

To sum up, PELNVs are useful in treating and preventing NAFLD, even though the exact mechanism is still unknown. PELNVs can be extracted from plants and produced at a large scale, making them advantageous for clinical therapy. However, it should be noted that PELNVs have less precise targeting abilities compared to MDEs, and they are not sourced from humans, which may affect their biocompatibility [111] (Table 2).

## The modification of EV

### Cargo packing and target drug delivery

EVs contain a variety of components, including mRNA, miRNAs, and other non-coding RNAs (ncRNAs) [112], that enable them to transport different cargoes. Besides, some exogenous therapeutic molecular drugs can also be transferred by EVs, including proteins, DNA, and small molecule drugs [35]. Some cells secrete exosomes when stimulated by external factors, and the cargoes of these exosomes can vary. Research has demonstrated that the cargoes carried in exosomes secreted by human umbilical vein cells (HUVECs) under normal conditions differ somewhat from those carried in exosomes secreted by HUVECs in a H_2_O_2_-induced senescence environment in vitro [113]. In addition, H2O2 could modulate exosomes biogenesis and autophagy flux in HUVECs, which provides new ideas for therapies for aging-related diseases [114]. EVs in their unmodified form typically exhibit low loading efficiencies. However, milk-derived EVs have the capability to load the chemotherapeutic drug paclitaxel (PAC) for oral delivery. It should be noted that the loading efficiency of this process is approximately 8% [115]. However, research demonstrated that doxorubicin (Dox) could be loaded by ginger-derived nanovectors (GDNVs) and modified with the targeting ligand folic acid (FA) for colon cancer therapy, and the loading efficiency was up to 95.9% [96]. Some exogenous drugs can be directly loaded into EVs by active (e.g., incubation of EVs and free drugs, incubation of the donor cells with free drugs) or passive (e.g., sonication, extrusion, freeze/thaw, electroporation, incubation with saponin, click chemistry, antibody binding) loading methods [116].

To solve the problem of targeting and minimizing side effects, engineering EVs becomes a promising approach for cell-free therapies. There are many methods for engineering EVs, such as surface engineering (e.g., genetic engineering, chemical modification) and membrane fusion [98].

The most commonly used method for genetic engineering is the use of plasmid vectors because EVs contain many transmembrane proteins (e.g., GPI, LAMP, LA and CD63, CD81, CD9). For instance, LAMP-2B, the membrane protein of dendritic cell-derived EVs, was combined with E7 peptide loaded with KGN as a therapy for osteoarthritis (OA) [117]. Liang et al. constructed pEGFP-CD63-Apo-AI and co-transferred with 293 T cell to generate targeting EVs [118].

There is limited research on chemical modification, but it is still possible to modify EVs through chemical methods. There are several methods for modifying surfaces. These include click chemistry, non-covalent modifications like multivalent electrostatic interactions, ligand-receptor interactions, hydrophobic interactions/membrane engineering, surface modification through aptamers, and modification by anchoring the CP05 peptide [119]. Since there is limited research on non-alcoholic fatty liver disease (NAFLD) in chemical modification extracellular vehicles (EVs), we will not delve into specifics.

About membrane fusion, EVs have a lipid bilayer membrane that allows them to fuse with cells that have the same membrane. Hepatocytes-derived EVs contain neutral ceramidase and sphingosine kinase 2 (SK2) and can fuse with hepatocytes, increasing intracellular sphingosine-1-phosphate (S1P). This can result in liver regeneration and cell proliferation [120].

### Artificial exosomes

Since cells only secrete a limited number of extracellular vesicles, researchers are exploring artificial exosomes as an alternative therapy method. Artificial exosomes are generally divided into three categories: nanovesicles (NVs), exosome mimics (EMs) and hybrid exosomes (HEs). As for the formation of NVs, there are several methods such as passing through membrane pores, microfluidic devices, nitrogen cavitation, sulfhydryl-blocking, exposure to alkaline solution [121].

Sometimes NVs produced by cell extrusion are also classified as EMs [122]. Yang et al. used a physical strategy to produce cell-derived EMs in sufficient quantities. They used a mimic extruder to extrude MCF-10A cells extruded through 10 μm, 5 μm, and 1 μm polycarbonate membranes. After ultracentrifugation, they obtained EMs with the same composition and performance as exosomes, and this method has a much higher yield than natural exosomes [123].

Another EMs are exosome-mimicking liposomes, a type of liposomes that may or may not contain specific proteins on the membrane [124]. Zhang et al. extracted lipids from edible ginger-derived nanoparticles to reassemble into ginger-derived nanovectors (GDNVs) modified with FA and loaded with Dox for colon cancer therapy, which showed a great capacity of tumor homing effect [96]. However, there are still some difficulties and problems in the production of liposomes such as complex, multi-step, not easy to large-scale production in the laboratory, specialized equipment and high cost [125].

As for HEs, Sato et al. confirmed that HEs can be produced through the use of membrane engineering and genetically modified techniques. In this technique, exosomes secreted from parent cells are fused with the membrane of synthetic liposomes by freeze–thaw method, which can be applied as an advanced drug delivery system [126]. Subsequently, Lin et al. applied HEs to encapsulate large CRISPR/Cas9 expression vectors, and they found that the loaded HEs could be endocytosed by MSCs to regulate targeted genes, which showed promise for curing genetic diseases [127].

## Conclusion

EVs hold promise as diagnostic biomarkers as well as therapeutic targets and drugs in liver disease. In this review, we summarize two roles for EVs in the treatment of NAFLD: as targets or as therapeutic agents. In addition, we have summarized some ways to improve EVs for better therapeutic outcomes. Because of the advantages of EVs such as low-toxic, low immunogenicity, biocompatibility, and targeting capability, EVs can serve as a targeted therapy for NAFLD. And as drug carriers, EVs should have the ability to carry large amounts of drugs. Therefore, EVs should be engineered for greater drug loading rates. However, as a drug, EVs are necessary to obtain in large quantities.

In fact, we still do not know how EVs work in the process of NAFLD, for example, specific molecular mechanisms such as biogenesis, cell entry, which require a large number of animal models for study. But EVs show the promising effects on obesity, NAFLD and other metabolic diseases. In addition, engineering EVs, acquisition of EVs, high volume clinical applications and EVs safety are still challenges. These challenges will provide new research directions in the fields of pharmacokinetics, toxicology and nanomedicine. The number of studies on EVs as a diagnostic as well as therapeutic tool for NAFLD is still growing, which will provide an important basis for future research on NAFLD.

## Data Availability

Not applicable.

## Abbreviations

T2DM: Type 2 diabetes mellitus
NAFLD: Non-alcoholic fatty liver disease
NASH: Non-alcoholic steatohepatitis
PNPLA3: Patatin like phospholipase domain containing 3
TM6SF2: Transmembrane 6 superfamily member 2
IFNγ: Interferon γ
TNFα: Tumor necrosis factor α
IL-6: Interleukin- 6
IR: Insulin resistant
TG: Triglyceride
HSCs: Hepatic stellate cells
MD: Mediterranean diet
AMPK/ULK1: Adenosine 5'-monophosphate (AMP)-activated protein kinase/ unc-51 like autophagy activating kinase 1
mTOR: Mammalian target of Rapamycin
EV: Extracellular vesicle
POH-POSS: Water-soluble multifunctional polyhydroxylated-polyhedral oligomeric silsesquioxane DRAM: DNA damage-regulated autophagy modulator
NAFL: Nonalcohol fatty liver
STOM: Stomatin
LMP: Lysosomal membrane permeabilization
MBVs: Multivesicular bodies
MVEs: Multivesicular endosomes
ESCRT: Endosomal sorting complex required for transport
ILVs: Intraluminal vesicles
RAB31: Ras-related protein in brain 31
TBC1D2B: The Rab22 GTPase-activating protein
ENO1: Alpha-Enolase 1
GPI: Glycosylphosphatidylinositol
HSPA5: Heat shock protein family A member 5
YWHAB: The 14–3–3 protein beta/alpha
CSF1R: Colony-stimulating factor receptor
CNTN1: The GPI-anchored protein contactin-1
GDNPs: Ginger-derived nanoparticles
PELNVs: Plant exosome-like nanovesicles
PA: Phosphatidic acid
MGDG: Monogalactosyldiacylglycerol
PC: Phosphatidylcholine
PI: Phosphatidylinositol
CHOL: Cholesterol
PS: Phosphatidylserine
PI: Phosphatidylinositol
CSF: Cerebral spinal fluid
DGC: Density-gradient centrifugation
ELVs: Exosome-like vesicles
DLS: Dynamic light scattering
NTA: Nanoparticle tracking analysis
TEM: Transmission electron microscopy
RPS: Resistive pulse sensing
AFM: Atomic force microscopy
HUVECs: Human umbilical vein cells
IMEX: Integrated magnetic-electrochemical exosome
PAC: Paclitaxel
Dox: Doxorubicin
FA: Folic acid
LAMP: Lysosome associated membrane protein
LA: Lactadherin
LAMP-2B: Lysosome associated membrane protein-2b
OA: Osteoarthritis
S1P: Sphingosine-1-phosphate
NVs: Nanovesicles
EMs: Exosome-mimics
HEs: Hybrid exosomes
WAT: White adipose tissue
BAT: Brown adipose tissue
UCP1: Uncoupling protein 1
CIT: Cold-induced thermogenesis
ADRB3: Beta3-adrenergic receptors
DIT: Diet-induced thermogenesis
BAT-Exos: BAT derived exosomes
eWAT: Epididymal WAT
FGF21: Fibroblast growth factor-21
ER: Endoplasmic reticulum
AKr1b7: Aldo–keto-reductase1b7
iPSCs: Induced pluripotent stem cells
ESCs: Embryonic stem cells
MSCs: Mesenchymal stem cells
MSC-Exos: MSC-derived exosomes
Huc-MSC: Human umbilical cord mesenchymal stem cells
HSTP1-Exos: HSTP1-modified Huc-MSC exosomes
aHSC: Activated hepatic stellate cell
hUCB-MSC: Human umbilical cord blood mesenchymal stem cell
ADSCs: Adipose-derived stem cells
ALI: Acute liver injury
MDEs: Mammalian cell-derived exosomes
BELNs: Blueberry-derived exosomes-like nanoparticles
HO-1: Heme oxygenase-1
FAS: Fatty acid synthase
ACC1: Acetyl-CoA carboxylase 1
Nrf2: Erythroid 2-related factor 2
FOX2: Forkhead transcription factor
GDV: Garlic-derived nanovesicles
H-VLNs: Component‒vesicle-like nanoparticles‒in honey
NLR: The nucleotide binding domain and leucine-rich repeat related
NLRP3: Pyrin domain containing 3
IEC: Intestinal epithelial cell
BMDMs: Bone marrow-derived macrophages
Ptgs2: Prostaglandin endoperoxide synthase 2
LOXs: Lipoxygenases
